# A Challenging Treatment Decision for a Rare Association: Case Report of Familial Turcot Syndrome With Fistulizing Crohn’s Disease

**DOI:** 10.3389/fped.2018.00083

**Published:** 2018-04-04

**Authors:** Montserrat Corbera-Hincapie, Genie L. Beasley

**Affiliations:** University of Florida Health, Gainesville, FL, United States

**Keywords:** Turcot syndrome, Crohn’s disease, familial adenomatous polyposis, ileal pouch-anal anastomosis, colonic adenocarcinoma

## Abstract

Turcot syndrome and fistulizing Crohn’s disease (CD) are two disease entities that are not usually associated with one another, particularly given the rarity of the former. This is a case of a pediatric patient with fistulizing CD treated with biologic therapy, who was later found to have Turcot syndrome. Management of this rare combination of diseases can present several challenges, as surgical options may be limited and chronic immunosuppression to treat CD may lead to accelerated progression of malignancy in Turcot syndrome. This unique case highlights the importance of weighing the risks and benefits involved in treating two disease entities that impact one another.

## Introduction

This case report illustrates the extremely rare association of Turcot syndrome and fistulizing Crohn’s disease (CD) in the same patient, which has only been described once in the literature (1). There are challenges surrounding diagnosis and most importantly management of patients who have this rare combination of diseases processes.

Familial adenomatous polyposis (FAP) is an autosomal dominant disorder caused by a mutation, usually nonsense or frameshift, of the adenomatous polyposis coli (APC) gene, a tumor suppressor gene located on chromosome 5q22. The disorder is characterized by hundreds to thousands of adenomatous polyps of the colon that carry a strong predisposition to develop into colorectal adenocarcinoma. Annual endoscopic screening should begin at age 10–12. Colonic adenomas first appear at an average age of 16 years, and once identified, colectomy is the only treatment recommended in order to prevent cancer. Nearly all affected patients will develop colorectal cancer by the sixth decade of life if prophylactic colectomy is not performed (2, 3, 4).

Turcot syndrome is a rare inherited disorder characterized by the association between adenomatous polyps in the colon with tumors of the central nervous system. About 150 cases of Turcot syndrome have been reported in literature (5). Turcot syndrome is thought to be a variant of FAP. There are two types of Turcot syndrome. Type I is characterized by the presence of few colonic polyps and glial tumors. Type II is autosomal dominant and has an association with FAP and central nervous system tumors, most commonly medulloblastoma (6).

Crohn’s disease is a chronic inflammatory disease of the gastrointestinal tract that is of unknown etiology and may affect any region from the mouth to the anus. The most common clinical manifestations are prolonged diarrhea with abdominal pain, weight loss, fever, and with or without gross gastrointestinal bleeding. In more severe cases, patients can develop fistula, or sinus tract formation (1). The most common fistula are enteroenteric, while other types include enterovesical and enterocutaneous (7). Management of CD varies based on severity, and exclusive enteral nutrition (EEN) is recommended as first-line therapy (8). Patients with mild to moderate disease who are not able to tolerate EEN are started on corticosteroids, aminosalicylates, or antibiotics. In patients with moderate to severe disease, corticosteroids or anti-tumor necrosis factor (TNF) can be used to induce remission. Maintenance therapy can include thiopurines, methotrexate, and/or anti-TNF therapy depending on severity (8, 9). Those with moderate to severe disease, particularly those with fistulizing CD, should be treated more aggressively with early introduction of anti-TNF therapy, which has been shown to have the best efficacy (10, 11).

## Case Report with Imaging

**Figure 1:**
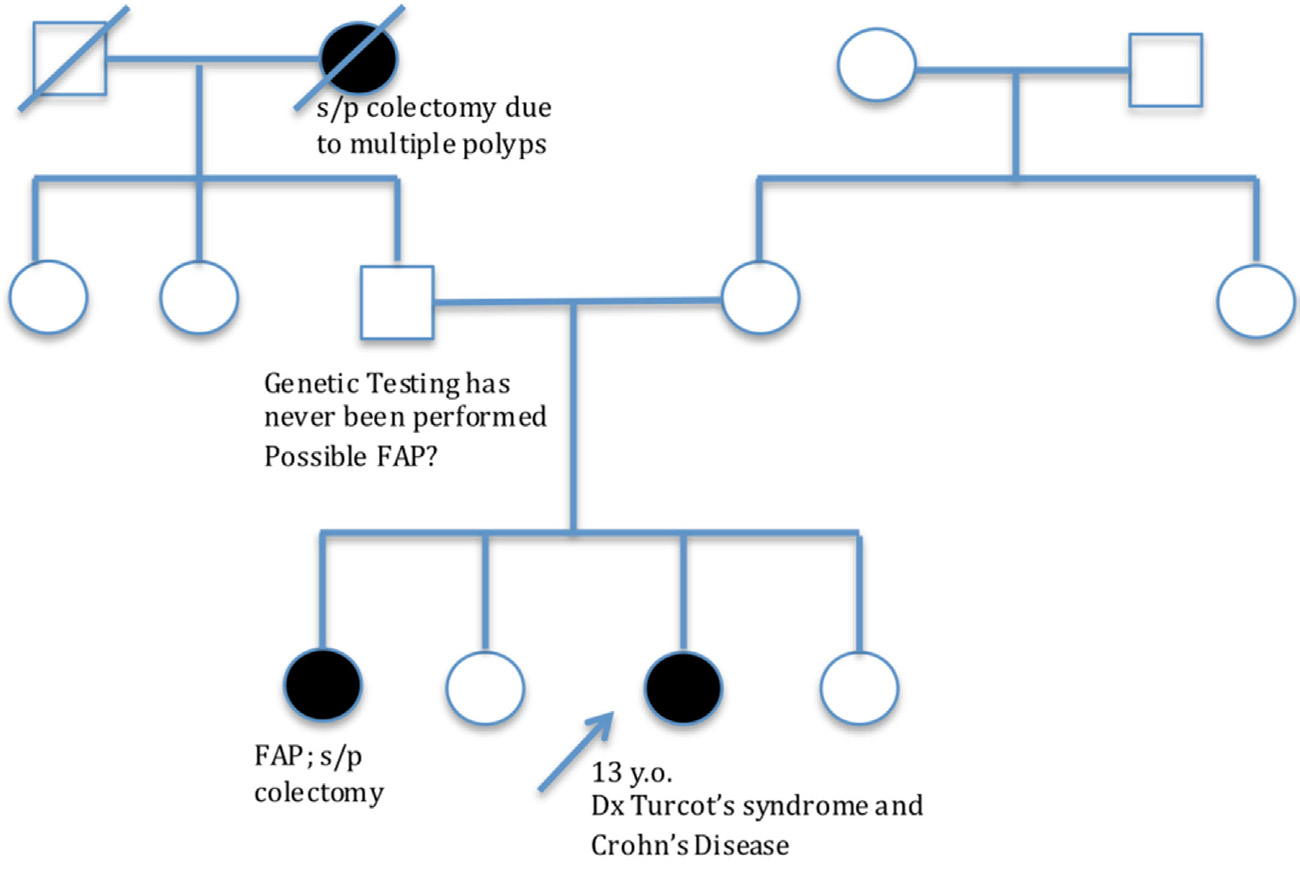
Family pedigree.

A 13-year-old female presented with a history of diarrhea with intermittent rectal bleeding, low-grade fever, tenesmus, anorexia, and weight loss. Of note, patient had a family history of genetically confirmed FAP, which included her sister and paternal grandmother (Figure 1). Given symptoms and family history, there was concern for polyposis syndrome and because of this, endoscopy and colonoscopy were performed. Anal skin tags were found on examination. There was no endoscopic evidence of polyposis; however, endoscopic exam did reveal pancolitis and histology was remarkable for granulomatous disease affecting both small and large bowel with active colitis. Diagnosis of CD was made. Patient was started on Mesalamine (Pentasa), and 6 months later, developed acute exacerbation of her CD requiring hospitalization. A perianal fistula was found on examination and confirmed by CT scan. She was started on EEN, but did not tolerate this so was transitioned to methylprednisolone and adalimumab. During this hospitalization, genetic testing for FAP was obtained; she is heterozygous for the c.3994dupA mutation in the APC gene. She carries an autosomal dominant mutation on the APC gene giving her a diagnosis of FAP.

**Figure 2:**
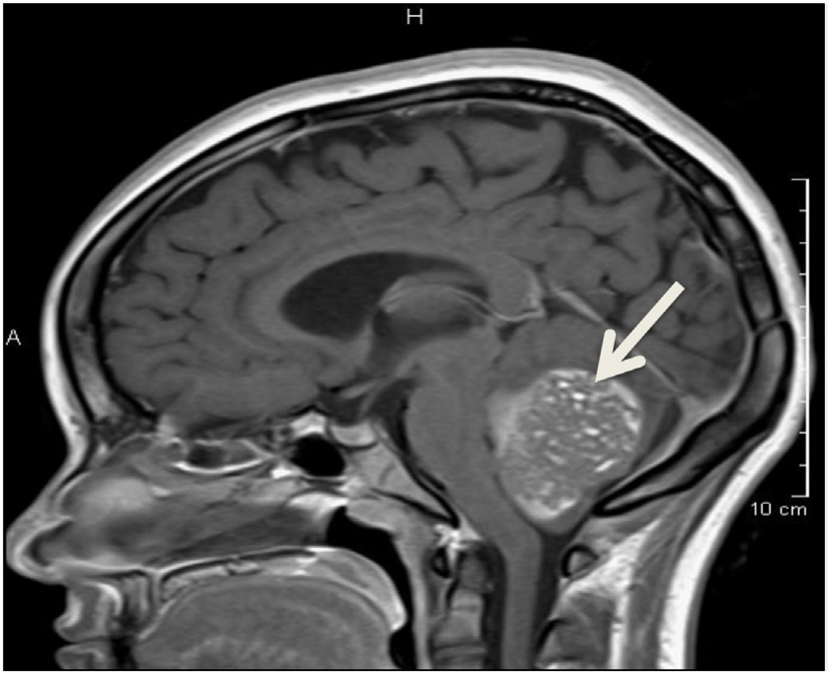
Large fourth ventricle mass consistent with medulloblastoma.

Almost a year after initial presentation, she developed headache, slurred speech, right arm weakness, and unsteady gait. She presented to the emergency department, where head CT scan showed a large fourth ventricle mass with hydrocephalus. Brain and spine magnetic resonance imaging was consistent with a large posterior fossa tumor with obstructive hydrocephalus (Figure 2), confirmed as medulloblastoma on histology. Genetic mutation consistent with FAP along with findings of medulloblastoma was diagnostic of Turcot syndrome. Patient underwent suboccipital craniectomy, gross total resection of the posterior fossa tumor, and placement of a right posterior occipital EVD shunt. Treatment included weekly vincristine for six doses and craniospinal radiation for 1 year, in which treatment with adalimumab was stopped. The patient developed posterior fossa syndrome following surgery, requiring intensive rehabilitation.

**Figure 3:**
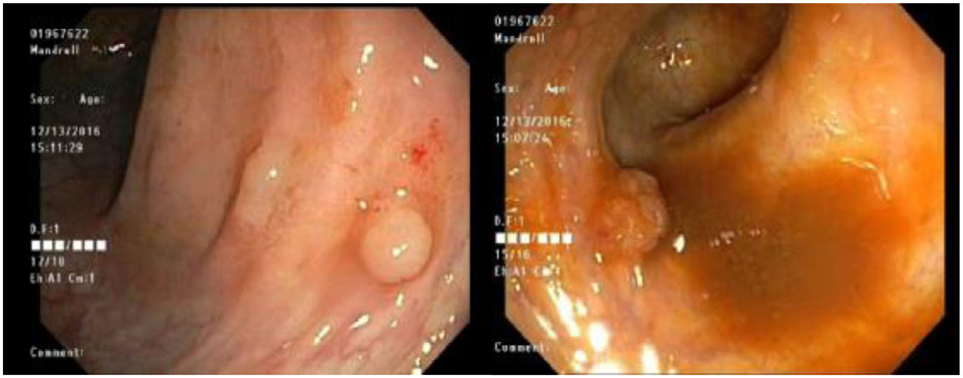
Surveillance colonoscopy showing multiple sessile polyps in the entire colon with histologic evidence of dysplasia.

Adalimumab was restarted upon completion of chemotherapy. Patient had a surveillance colonoscopy and endoscopy performed 1 year after diagnosis of FAP and was remarkable for multiple small duodenal adenomatous polyps and several tubular adenomas in colon (Figure 3) with no dysplastic changes. Endoscopic exam 3 years after FAP diagnosis was remarkable for numerous duodenal and colonic adenomas and low-grade dysplasia in the sigmoid colon and rectum.

## Discussion

The prevalence of FAP in the United States is about 1:10,000 to 1:30,000 and accounts for less than 1% of all colorectal cancers. The prevalence of FAP and CD is unknown and has only been reported once in the literature (1). The association of Turcot syndrome with fistulizing CD, as seen in this patient, raises several questions about management. It is unclear if chemotherapy or chronic immunosuppression accelerates the process of polyp development or dysplastic changes. Hence, this patient underwent side-viewing endoscopy early in her disease course (12) to monitor for dysplastic changes in her duodenum. This patient was treated with immunosuppressive agents, including adalimumab, as well as chemotherapy for medulloblastoma, prior to development of polypoid low-grade dysplasia in her colon. Her development of dysplasia begs the question of possible acceleration of polyp formation and dysplasia when exposing a patient with a known APC mutation to immunosuppressive or chemotherapy. The other question that is raised in a patient like ours with fistulizing perianal disease is long-term management once polyposis of the colon is discovered. To prevent development of colon cancer, the patient will need to undergo total colectomy; however, there needs to be careful consideration of whether the patient would be a candidate for a J-pouch construction and ileal pouch-anal anastomosis (IPAA), given her history of perianal fistula, versus permanent ileostomy.

### Role of Immunosuppressive Therapy in Acceleration of Dysplasia

Colorectal cancer represents the third most common malignancy and the leading cause of cancer death. It is estimated that fewer than 10% of adenomas develop into cancers; however, more than 95% of colonic adenocarcinomas develop from adenomas (13). Survivors of other cancers have shown to have increased risk of secondary malignancies. There has been growing evidence to implicate factors, such as immunosuppressive therapy, previous radiation exposure, chemotherapy, and genetic predisposition, in rapid progression of advanced adenocarcinomas. Our patient not only carries the genetic mutation predisposing her to cancer formation but also underwent treatment with intracranial radiation and systemic chemotherapy for her medulloblastoma, along with years of immunosuppression with biologic therapy for CD, placing her at high risk for accelerated progression of secondary malignancies (14). After radiation therapy (RT), interval development of secondary malignancy is dependent on the radiation dose to the organ treated directly and typically occurs between 10 and 15 years post-radiotherapy. The evidence should encourage use of lowest possible dose of RT, along with continued cancer surveillance. After receiving chemotherapy, especially alkylating agents such as procarbazine, there is thought to be a ninefold increased risk of subsequent colorectal cancer. There is emerging evidence that there is an elevated risk of subsequent neoplasms in patients with solid organ transplants who undergo chronic and lifelong immunosuppressive therapies. It has become clear that immune alterations have an impact on the development of neoplasms.

### Medication Management Considerations

Given the extensive literature data indicating that risk for malignancy is higher is cancer survivers and in the setting of immune suppression, it is imperative that thoughtful management decisions should be considered in patients such as ours. When our patient was found to have perianal fistula, EEN was initiated. This form of treatment, which involves liquid diet and exclusion of normal diet, is safe, long term, and provides effective management of CD with no adverse side effects (15). Data support use of EEN for patients with small bowel disease, while this treatment’s effectiveness in patients such as ours, with mainly colonic disease is still unknown (8). Recently, there have been small reports suggesting EEN may be an effective and low-risk treatment for fistulizing CD (16), though long-term compliance with diet can be difficult. Our patient was unable to follow the diet as directed, and instead was treated with biologic agents for her penetrating disease. In doing so, this may have increased her risk for development of malignancy.

### Ileostomy Versus J-Pouch Construction and IPAA: Surgical Management Dilemma

Restorative proctocolectomy (RPC) and IPAA, also referred to as J-pouch construction, have been the procedures of choice for patients with FAP. In patients like ours with fistulizing CD, an ileo-anal J-pouch has been considered to be contraindicated by some due to risk for pouch complications and failure. Complications include development of chronic pouchitis and fistulizing disease involving the pouch (17). In a meta-analysis, Reese et al. compared outcomes of RPC and IPAA in patients with ulcerative colitis, CD, and indeterminate colitis. Patients with CD have an increased rate of anastomotic strictures, pouch failure, and pouch inflammation. In patients such as ours with limited surgical options, there should be awareness that although performing restorative procedures is a key feature in maintaining better quality of life for some, creation of a permanent diverting ileostomy may be a better option for prevention of complications.

## Conclusion

The rare occurrence of CD and Turcot syndrome in one patient illustrates the need to balance the challenges associated with treatment of each disease. The management is particularly challenging since medical treatment and surgical options must be carefully considered, as immunosuppressive therapy, previous radiation exposure, and genetic predisposition have potential to increase cancer risks. Further studies such as efficacy of long-term EEN as primary therapy for CD are necessary to determine optimal management in these patients, how treatment of one disease affects the other in the long term and whether a stricter surveillance program is warranted.

## Ethics Statement

I confirm that written informed consent was obtained from patient for publication of the case details.

## Author Contributions

MC-H is the primary author. GB is a co-author and editor.

## Conflict of Interest Statement

The authors declare that the research was conducted in the absence of any commercial or financial relationships that could be construed as a potential conflict of interest.
